# Production and characterization of rNGFSP: a recombinant fusion immunogen eliciting dual anti-NGF and anti-Substance P therapeutic antibodies for Degenerative Joint Disease

**DOI:** 10.1016/j.btre.2026.e00946

**Published:** 2026-01-12

**Authors:** Valentina Varela, Monique Costa, Cecilia Maciel, Joaquín Barbeito, Exequiel E. Barrera, Erica Gutierre, Agustín Correa, Melania Elgue, Sebastián Carrasco, Magdalena Domínguez Larrosa, María Pereira, Josefina Correa, Nadia Crosignani, Joseph S. Beckman, Luis Barbeito, Emiliano Trias

**Affiliations:** a Xeptiva Therapeutics, Montevideo 11.300, Uruguay; b Neurodegeneration Lab, Institut Pasteur de Montevideo, Montevideo 11.400, Uruguay; c Instituto de Histología y Embriología de Mendoza (IHEM), Universidad Nacional de Cuyo, CONICET, Mendoza, Argentina; d Unidad de Farmacología, Departamento de Clínicas y Hospital Veterinario, Facultad de Veterinaria, UdelaR, Montevideo 13.000, Uruguay; e Protein Engineering Unit, Institut Pasteur de Montevideo, Montevideo 11.400, Uruguay; f Unidad de Clínica y Cirugía de Pequeños Animales, Facultad de Veterinaria, UdelaR, Montevideo 13000, Uruguay; g e-MSion Inc., 2121 NE Jack London Street, Corvallis, OR 97330, United States; h Linus Pauling Institute, Department of Biochemistry and Biophysics, Oregon State University, 97331, United States

**Keywords:** Chronic pain, Osteoarthritis, NGF, Substance P, Recombinant fusion antigen, Self-antigen vaccines

## Abstract

Anti-NGF monoclonal antibodies have recently been approved for treating degenerative joint disease, including osteoarthritis pain, in dogs and cats. However, their widespread use is limited by high cost and the requirement for repeated injections. Nerve Growth Factor and Substance P play central roles in the initiation and maintenance of inflammation and chronic pain in OA. There is a pressing need for new, safe, cost-effective therapies that target the underlying mechanisms of OA chronic pain. Here, we designed and produced a novel recombinant fusion protein, termed rNGFSP, which functions as an immunogen due to its unique molecular structure combining amino acid sequences from NGF and SP in a non-native conformation. When formulated and administered as a vaccine, rNGFSP elicits dual anti-NGF and anti-SP therapeutic antibodies in the host. rNGFSP was produced in *E. coli* and purified from inclusion bodies using metal affinity chromatography under denaturing conditions. Mass spectrometry confirmed the expected molecular weight (17.5 kDa) and preserved amino acid sequence. Structural prediction using Alphafold2 revealed rNGFSP presented a non-natural folding, but a preserved NGF core and a flexible SP tail, supporting antigenic presentation. Vaccination of mice, rabbits, horses, and dogs, showed that rNGFSP elicited cross-reactive IgG antibodies against the native conformations of NGF and SP. Furthermore, immunoglobulins elicited in vaccinated dogs neutralized the biological activity of NGF and SP in cell cultures, suggesting a therapeutic potential. These findings support rNGFSP as a promising vaccine candidate simultaneously targeting endogenous NGF and SP species, providing a cost-effective alternative to monoclonal antibodies.

## Highlights

• A novel recombinant fusion immunogen (rNGFSP) was designed to induce antibodies against both NGF and SP, key mediators of inflammation and chronic pain in OA.
• rNGFSP was expressed in *E. coli*, purified under denaturing conditions, and structurally characterized by mass spectrometry and *in silico* modeling.
• Immunization with rNGFSP in mice, rabbits, horses, and dogs, was safe and elicited robust IgG responses that neutralized NGF and SP biological activities in cell cultures.
• rNGFSP administered to mammals as a vaccine represents a cost-effective and scalable biotechnological platform for active immunotherapy for the treatment of degenerative joint disease.

## 1 Introduction

Degenerative joint disease (DJD), including osteoarthritis (OA), is a highly prevalent condition in veterinary medicine, particularly affecting aging dogs and cats. These progressive disorders are characterized by cartilage degradation, joint inflammation, and persistent pain, leading to reduced mobility and quality of life [[1], [2], [3], [4]]. Among the molecular drivers of DJD-associated inflammation and pain, Nerve Growth Factor (NGF) and Substance P (SP) have been identified as key synergistic mediators [[1], [2], [3], [4]].

NGF secreted by various cell types in response to injury contributes to peripheral pain sensitization by binding to the high-affinity TrkA receptor on nociceptive sensory neurons, lowering their activation threshold and intensifying pain perception [5]. In parallel, NGF stimulates these neurons to synthesize and release pro-inflammatory neuropeptides, particularly SP [6,7]. In turn, NGF-sensitized nociceptors in OA joints release SP through antidromic axonal reflexes which acts on various cellular targets within the joint microenvironment, such as mast cells, endothelial cells, synoviocytes, and macrophages. Through activation of neurokinin-1 (NK1R) and MRGPRX2 receptors, SP triggers vasodilation, immune cell recruitment, degranulation of mast cells, and the release of inflammatory mediators [1,4,[8], [9], [10]].

The reciprocal interactions between NGF and SP contribute to a self-amplifying neuroimmune loop known as neurogenic inflammation that perpetuate peripheral sensitization, synovial inflammation, and progressive cartilage degradation [1,[11], [12], [13], [14], [15]]. Elevated levels of NGF and SP in the synovial fluid of OA patients, positively correlated with proinflammatory cytokines, suggesting the relevance of this neurogenic mechanism in chronic joint inflammation [1,3,4,15]. Therefore, NGF and SP emerge as key mediators underlying OA chronic pain and possibly disease progression, providing a compelling rationale for therapeutic interventions that simultaneously disrupt this neuroimmune feedback loop.

Despite evidence supporting the significance of the NGF–SP axis in OA, currently available immunotherapeutic strategies focus exclusively on targeting NGF, primarily through anti-NGF monoclonal antibodies Bendivetmab and Frunevetmab, recently approved for use in dogs and cats, respectively [[16], [17], [18], [19], [20]]. These monoclonal antibodies have demonstrated efficacy in reducing hyperalgesia and behavioral indicators of pain in pets with OA. Recently, innovative vaccine strategies employing virus-like particles conjugated with NGF have shown promise in murine models by eliciting polyclonal anti-NGF antibody responses, significantly reducing OA pain behaviors [21,22]. Despite these advances, widespread adoption of monoclonal antibody therapies in veterinary medicine remains limited [23] due to their high cost and necessity for repeated chronic injections. Thus, there is a need for broader-acting therapeutic strategies that include targeting SP, for which no established or experimental immunotherapies currently exist for OA inflammation and pain. We have hypothesized that the simultaneous targeting of the NGF-SP axis through immunological strategies offers a promising multimodal approach for treating OA chronic pain and inflammation.

In this study, we designed and produced a novel recombinant fusion protein, rNGFSP, intended as an immunogen for therapeutic vaccination. rNGFSP combines amino acid sequences from NGF and SP in a non-native conformation, lacking biological activity due to its expression in prokaryotic cells and purification under denaturing conditions from inclusion bodies. Despite the absence of native folding, rNGFSP demonstrated strong immunogenicity across multiple host species when administered as a vaccine. Immunization with rNGFSP induced IgG antibodies cross-reactive with the native conformations of both NGF and SP, which also exhibited neutralizing activity in established cell-based functional assays of the neuropeptides. These results support the use of rNGFSP as a novel vaccine-based strategy for targeting neurogenic inflammation and chronic pain in companion animals.

## 2 Material and methods

### 2.1 Expression of recombinant fusion immunogen rNGFSP

**Fig. 1:**
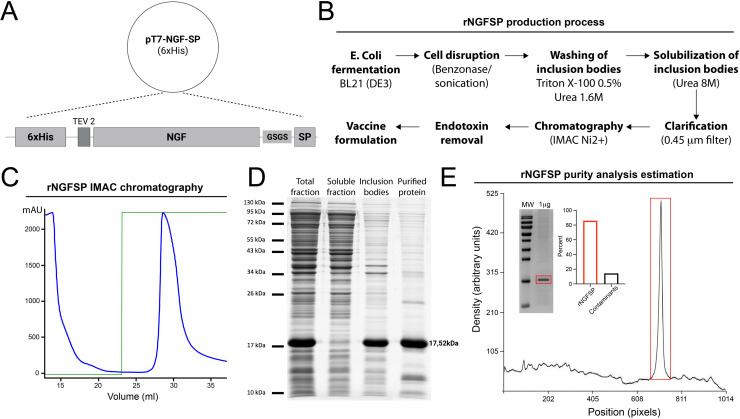
**Expression, purification, and characterization of the recombinant fusion protein rNGFSP.** A) Schematic representation of the rNGFSP expression construct cloned into the pT7 vector, including an N-terminal 6 × His tag, a TEV protease cleavage site, the NGF domain, and a C-terminal Substance P sequence, linked by a flexible GSGS linker. B) Workflow diagram of rNGFSP production in *Escherichia coli* BL21 (DE3). Following cell disruption (Benzonase and sonication), inclusion bodies (IBs) were washed with 0.5% Triton X-100 and 1.6 M urea, solubilized in 8 M urea, clarified through a 0.45 μm filter, and purified by immobilized metal affinity chromatography (IMAC, Ni²⁺). Endotoxin removal was performed prior to final vaccine formulation. C) IMAC chromatographic profile of rNGFSP purification. The blue line represents protein absorbance (mAU) during elution, and the green line indicates the imidazole pulse (10 mL) used to recover the bound protein. D) SDS-PAGE analysis showing the stepwise purification of rNGFSP. Lanes correspond to: total protein fraction (lane 1), soluble fraction (lane 2), inclusion bodies (lane 3), and purified rNGFSP (lane 4). The expected molecular weight of rNGFSP is ∼17.5 kDa. E) Purity analysis of rNGFSP by densitometric quantification of the SDS-PAGE gel. The main band corresponds to rNGFSP, while residual contaminants represent a minimal percentage of the total protein content.

The recombinant fusion protein rNGFSP was expressed in *E. coli* BL21 (DE3) cells. Chemically competent BL21 (DE3) cells (Thermo Fisher, EC0114) were transformed with the pT7 expression plasmid [24] encoding a codon-optimized rNGFSP gene, which includes an N-terminal polyhistidine tag (Fig. 1), following the manufacturer’s instructions. Transformed colonies were cultured overnight at 37 °C in Luria-Bertani (LB) medium supplemented with 100 µg/mL ampicillin (Sigma-Aldrich, A0166) under agitation at 220 rpm.

The rNGFSP construct consists of an N-terminal His tag, a central domain corresponding to canine NGF, and a C-terminal monomeric peptide derived from SP, linked by a flexible linker that codes to a GSGS peptide, with the following amino acid sequence:

MRGSHHHHHHGSGSENLYFQGSSSSHPVFHRGEFSVCDSVSVWVGDKTTATDIKGKEVMVLGEVNINNSVFKQYFFETKCRDPTPVDSGCRGIDSKHWNSYCTTTHTFVKALTMDGKQAAWRFIRIDTACVCVLSRKAGRRAGSGSRPKPQQFFGLM

To evaluate the reproducibility and efficiency of the rNGFSP, three independent batches were produced using an identical expression protocol. For high-yield expression, 10 mL of an overnight *E. coli* culture harboring the rNGFSP expression plasmid were used to inoculate 1 L of Terrific Broth (TB). Cultures were incubated at 37 °C with continuous agitation at 220 rpm until the optical density at 600 nm (OD₆₀₀) reached 2.0, indicating mid-log phase growth. Protein expression was then induced by the addition of 1 mM isopropyl β-D-1-thiogalactopyranoside (IPTG; Euromedex, EU0008-B), followed by continued incubation under the same temperature and agitation conditions for 4 h to allow recombinant protein accumulation.

Cells were harvested by centrifugation at 10,000 × *g* for 10 min at 4 °C and resuspended in lysis buffer consisting of 30 mM Tris–HCl (pH 8.0), 150 mM NaCl, and 0.5 mg/mL lysozyme. The bacterial suspension was lysed by sonication using a pulse protocol (1:40 min total; 20 s ON, 1 min OFF; 50% amplitude), followed by centrifugation at 20,000 × *g* for 40 min at 4 °C. The resulting pellet, containing inclusion bodies, was subjected to two sequential washing steps: first with Washing Buffer I (50 mM Tris–HCl, pH 8.0; 50 mM NaCl; 0.5% Triton X-100; 1.5 mM β-mercaptoethanol; 1.6 M urea), and subsequently with Washing Buffer II (30 mM Tris–HCl, pH 8.0; 150 mM NaCl). Each wash was followed by centrifugation at 20,000 × *g* for 20 min at 4 °C.

### 2.2 Immobilized metal affinity chromatography (IMAC)

The final washed pellet was solubilized in 20 mM Tris–HCl (pH 8.0), 500 mM NaCl, and 8 M urea. Solubilized protein was purified by IMAC at room temperature using a 5 mL HisTrap™ Chelating HP column (GE Healthcare, Cytiva, 17-5248-02) pre-charged with Ni²⁺. The column was equilibrated with a solubilization buffer prior to loading the sample. Non-specifically bound proteins were removed by washing with five column volumes of the same buffer. The rNGFSP fusion protein was eluted in three 10 mL fractions using an elution buffer containing 20 mM Tris–HCl (pH 8.0), 500 mM NaCl, 500 mM imidazole, and 8 M urea. Eluted fractions were assessed by SDS-PAGE, and those containing rNGFSP were pooled and dialyzed extensively to remove imidazole.

### 2.3 Endotoxin removal

To ensure low endotoxin levels suitable for in vivo administration, the purified rNGFSP was incubated with endotoxin removal magnetic beads (Miltenyi Biotec, #130-093-657) according to the manufacturer’s instructions. Endotoxin quantification was performed using the Pierce™ LAL Chromogenic Endotoxin Quantitation Kit (Thermo Fisher, #88282), with values maintained within acceptable thresholds for preclinical immunization.

### 2.4 Protein quantification

Total protein concentration was determined by the Pierce 660 assay (Pierce, #22660) using bovine serum albumin as the standard. The standard curve and all measurements were conducted in 20 mM Tris–HCl (pH 8.0), 500 mM NaCl, and 8 M urea.

### 2.5 Estimation of rNGFSP purity

The purity of rNGFSP was assessed before vaccine formulation by SDS-PAGE followed by Coomassie Brilliant Blue staining. Samples were denatured, loaded onto a 15% polyacrylamide gel, and electrophoresed under reducing conditions. Protein bands were visualized, and the relative purity was estimated by densitometric analysis using ImageJ software, expressed as the percentage of the target band intensity relative to total protein content.

### 2.6 DNA quantification by fluorometry

DNA quantification was performed using the Qubit™ DNA quantification kit (code 281120) following the manufacturer’s instructions. A total of three independent batches were analyzed. For each measurement, 10 µL samples were used. Fluorescence was measured using a Qubit fluorometer, and DNA concentrations were calculated using the kit-provided standards.

### 2.7 Mass spectrometry analysis

The rNGFSP protein was characterized by mass spectrometry (MS) as described previously [[25], [26]]. To confirm the intact mass and the amino acid sequence of the purified rNGFSP antigen, 10 µL of a 16 mg/mL rNGFSP solution in 8 M urea, 500 mM NaCl, and 20 mM Tris buffer was precipitated in 500 µL of ammonium acetate (NH₄Ac) aqueous solution (pH 7) at room temperature. The sample was centrifuged, and the pellet was washed twice with the same aqueous solution. After removal of the supernatant, the precipitate was reconstituted in 500 µL of 5% acetic acid (HAc) by vortex mixing at room temperature. The resulting solution was introduced into an Agilent 6545XT mass spectrometer at a flow rate of 20 µL/min and analyzed by MS1 (intact mass) and also top-down MS/MS using electron capture dissociation (ECD) fragmentation. Data processing was performed using ExDviewer software for Top Down characterization and Agilent MassHunter Bioconfirm software for MS1 Deconvolution.

### 2.8 In silico structural modeling and molecular dynamics simulations

The rNGFSP construct including the polyhistidine tag was modeled using AlphaFold2.1. using the predicted amino acid sequence shown above. From the set of predicted structures, the three top-ranked models based on confidence metrics (pLDDT) were selected as initial conformations for further conformational refinement via molecular dynamics simulations.

The selected rNGFSP models were subjected to atomistic molecular dynamics (MD) simulations to evaluate structural flexibility and conformational stability in solution. Each structure was simulated for 1 μs in triplicate using the GROMACS MD engine, version 2020.4. Simulations were conducted in an explicit solvent environment of 8 M urea and 500 mM NaCl, reflecting the physicochemical conditions used during antigen formulation. The Amber ff14SB force field was applied for all simulations, according to Abraham et al. [27] and Maier *et al*. [28].

To identify dominant structural conformations, trajectory clustering was performed using the GROMOS algorithm on the last 0.1 μs of each simulation. This allowed selection of the most populated conformational clusters representative of the equilibrium behavior of rNGFSP.

### 2.9 Vaccine formulations

Each dose was prepared in sterile conditions and in a solution of 20 mM Tris pH 8, 500 mM NaCl and 4 M Urea. Different formulations were prepared for different species as described in Table 1. All formulations contained the vaccinal antigen rNGFSP and different concentrations of Montanide Gel 01 PR (SEPPIC) in a total volume of 100 μL to 2 mL. All vaccine preparations were freshly prepared under sterile conditions prior to administration.

**Table 1:** Description of the different vaccine formulations used for each immunized species.

	Mice (*n* = 4)	Rabbits (*n* = 5)	Horses (*n* = 3)	Dogs (*n* = 6)
rNGFSP	20 µg	70 µg	700 µg	70 µg
Adjuvant concentration	1%	0.5%	5%	1%
Final volume	0,1 ml	2 ml	1 ml	1 ml

### 2.10 Safety assessment in mice

Preclinical safety of the rNGFSP vaccine candidate was evaluated in 6 months-old C57BL/6 mice of both sexes. Vaccine composition in mice was 20 µg of rNGFSP with 1% of adjuvant Montanide™ Gel 01, in a total volume of 100 µL per animal. The immunization schedule consisted of a priming dose on Day 0 followed by boosters on Days 14, 28, and 42. A total of two experimental groups (*n* = 3–5 mice per group) were established receiving either the rNGFSP formulation or excipient control (vaccine vehicle).

Hematological parameters were assessed through submandibular blood samples collected prior to immunization, seven days after each booster dose, and fourteen days after the final booster dose. The blood samples were incubated at room temperature for 2 h and then centrifuged at 3000 x *g* for 10 min at 4 °C to separate the serum. Hematological analyses were performed using a Mindray BC-500 Vet veterinary hematology analyzer. For assessment of hepatic and renal function, additional blood samples were obtained at baseline, 48 h after each immunization, and fourteen days post-final dose. Biochemical analyses were conducted using the Pointcare V2 system, measuring markers of liver integrity and kidney function.

Throughout the immunization protocol, animals were monitored daily by trained personnel for general health and behavior. Specific attention was given to changes in body temperature, clinical signs of local inflammation at the injection site, regional lymphadenopathy, and overall condition.

### 2.11 Immunization of rabbits, horses and dogs

New Zealand adult rabbits (1.6–1.8 kg, *n* = 5) were housed in individual cages in a farm approved animal facility in Canelones, Uruguay. Horses (3- to 5-year-old colts) were client-owned animals (*n* = 3), housed in a stable located in Canelones, Uruguay. Dogs (mixed-breed, 1–3 year old, 6–12 kg, *n* = 6), were recruited from a shelter in the Montevideo region (Uruguay) and housed in kennels containing 2 to 5 animals each. All animals were clinically healthy, free of detectable systemic diseases, not undergoing any medical treatments, and presented normal hematological and biochemical serum parameters (Data not shown). All animals received immunizations with the formulations as described in Table 1. All administrations were subcutaneously injected at distinct dorsal cervical sites (within the neck triangle), which were cleansed and sterilized with 70% alcohol prior to vaccine administration. Animals were fasted overnight prior to immunization and blood sampling, but regular feeding was resumed immediately after the procedures.

In all three species the immunization protocols included four doses (a priming dose followed by three boosters) administered at two-week intervals, followed by a 14-day recovery period (Fig. 3). Pre-immune sera were collected prior to the first immunization to establish baseline antibody titers.

### 2.12 Determination of antibodies against the vaccine antigen

Immunogenicity of rNGFSP was evaluated by measuring antigen-specific IgG antibodies generated in response to immunization with rNGFSP in different animal models using an optimized indirect ELISA. For mice and rabbits, high-binding 96-well microplates (Costar high binding, #3590, Corning Incorporated, USA) were coated overnight at 37 °C with rNGFSP (2.5 μg/mL) diluted in 50 mM carbonate buffer (pH 9.6), then blocked with PBS containing 1% gelatin. Serum samples, diluted 1:1000 in PBS with 0.05% gelatin and 0.1% Tween-20, were incubated in duplicate for 1 h at 37 °C. After washing with PBS containing 0.1% Tween-20, bound IgG was detected using horseradish peroxidase (HRP)-conjugated goat anti-mouse IgG antibody (Abcam, ab6789) or anti-rabbit IgG (Abcam, ab6721).

For horses and dogs, a similar ELISA protocol was applied with modifications to the blocking and dilution buffers. Plates were also coated with rNGFSP (2.5 μg/mL) and incubated overnight at 37 °C, followed by blocking with PBS containing 3% non-fat dry milk. Serum samples, diluted 1:1000 in PBS with 3% milk and 0.5% Tween-20, were incubated in duplicate for 1 hour at 37 °C. After washing with PBS containing 0.5% Tween-20, bound IgG was detected using HRP-conjugated goat anti-dog IgG antibody (Abcam, ab112852) or anti-horse IgG (Abcam, ab6921). Detection in all assays was carried out using 3,3′,5,5′-tetramethylbenzidine (TMB) substrate. The enzymatic reaction was stopped with 1 M H₂SO₄, and absorbance was measured at 450 nm using a Multiskan MS plate reader (Thermo Fisher). Antibody response kinetics were assessed by monitoring the increase in O.D. values during the specified time points.

### 2.13 Determination of antibodies cross-reactive to native NGF and SP

To assess the humoral response against the native conformation of NGF and SP in dogs, rabbits and horses, indirect ELISAs were performed as described above. The coating was performed with either dimeric recombinant NGF (ProSpec, CYT579, 2.5 μg/mL, overnight at 4 °C) or custom-synthesized BSA-conjugated SP (GeneScript, 8 μg/mL, overnight at 4 °C). Serum samples were diluted at 1:1000 for NGF and 1:100 for SP in dogs and horses, while in rabbits, dilutions of 1:250 for NGF and 1:50 for SP were used.

### 2.14 NGF-TrkA binding assay

A competitive ELISA was performed to evaluate the ability of IgG antibodies elicited by rNGFSP vaccination to inhibit NGF binding to the extracellular domain of the TrkA receptor. This assay assessed the neutralizing potential of immune sera by measuring interference with NGF-TrkA interaction [29]. High-binding 96-well microplates (Costar #3590, Corning) were coated overnight at 4 °C with 2 μg/mL recombinant human TrkA (extracellular domain, source: MyBioSource #MBS8123129) diluted in carbonate buffer (50 mM, pH 9.6) in a total volume of 100 μL per well. Plates were washed three times with PBS-T (PBS with 0.1% Tween-20) and then blocked with 1% BSA in PBS (200 μL per well) for 1 h at 37 °C. For the neutralization step, biotinylated NGF (ProSpec, CYT-579, final concentration 0.5 μg/mL) was pre-incubated in PBS with dog sera diluted 1/10 in PBS (pre-immune or hyperimmune) for 1 h at 37 °C in a final volume of 100 μL. Control wells included biotinylated NGF alone (no serum) and NGF pre-incubated with non-immune sera. After incubation, the mixtures were added to the TrkA-coated wells and incubated for 1 h at 37 °C to allow competition for TrkA binding. Plates were washed three times with PBS-T, then incubated with streptavidin-HRP (1:10,000 dilution in PBS, 100 μL/well) for 1 h at 37 °C. After three additional washes, binding was revealed using TMB substrate (Thermo Fisher). The enzymatic reaction was stopped with 1 M H₂SO₄, and absorbance was measured at 450 nm using a Multiskan MS plate reader (Thermo Fisher). Inhibition of NGF binding to TrkA by immune sera was expressed as a reduction in OD450 values relative to the control condition with NGF alone.

### 2.15 TF1 cell proliferation assay

The TF1 human erythroleukemic cell line (ATCC #CRL-2003), which expresses the TrkA receptor, was used to assess the NGF-like activity of rNGFSP and the neutralizing capacity of anti-NGF antibodies, following a protocol adapted from [30]. Cells were seeded at 15,000 cells/well in 96-well plates in 150 μL RPMI-1640 supplemented with 10% FBS, without GM-CSF. After 1 h incubation at 37 °C, treatments (50 μL/well) were added. Conditions included: NGF (10 ng/mL), rNGFSP (400 ng/mL), NGF pre-incubated with 1:10 diluted pre-immune or hyperimmune dog sera, and appropriate controls. After 48 h incubation, 20 μL CCK-8 reagent (Abcam, ab228554) was added to estimate cell number, followed by 24 h incubation. Absorbance at 460 nm was measured using a Varioskan Lux reader (Thermo Fisher). Proliferation was expressed as relative absorbance; inhibition by immune sera was interpreted as NGF neutralization.

### 2.16 SP-induced mast cell degranulation assay

A mast cell degranulation assay was used to evaluate the SP-like activity of rNGFSP and the neutralizing capacity of IgGs from vaccinated dogs. The assay quantifies mast cell degranulation by detecting the binding of fluorescent avidin to heparin exposed on the external surface of the plasma membrane following granule release [31]. Primary murine bone marrow-derived mast cells (BMMCs) from adult C57BL/6 mice were generated as described by Swindle *et al*., 2000 [32], cultured in RPMI-1640 with 15% FBS, 20 ng/mL IL-3, and 20 ng/mL SCF (Miltenyi Biotec), and used after 4–6 weeks following confirmation of maturation by flow cytometry. Degranulation assays to assess SP-like activity of rNGFSP were performed using 10 µM rNGFSP solubilized in PBS as compared to 10 µM of SP. To assess the neutralization capacity of IgG produced in dogs, total IgG was purified from the pooled sera of dogs immunized with rNGFSP using NAb™ Protein G Spin Columns (Thermo Scientific, Cat# 89953), following the manufacturer’s instructions. Assays were performed by incubating 1 × 10⁵ BMMCs in 250 µL stimulation buffer (RPMI + 70 mM NaCl) for 30 min at 37 °C with: (i) PBS (control), (ii) compound 48/80 (MRGPR-receptor agonist, 10 µg/mL), (iii) SP conjugated to BSA conjugate (0.2 mg/mL), or (iv) SP-BSA pre-incubated 30 min at room temperature with 0.5 mg/mL IgGs purified from pools of dog sera before and after immunization (D0 versus D56). After stimulation, cells were centrifuged (1000 × g, 10 min, 4 °C), resuspended in 150 µL PBS, and stained with 2 µg/mL Avidin-FITC (Thermo Scientific, #A821) for 20 min at room temperature. Samples were fixed in 1% PFA and analyzed by flow cytometry (Attune™ NxT, Thermo Fisher) and laser scanning Zeiss LSM 800 confocal microscope. Degranulation was quantified by the increase in Avidin-FITC fluorescence.

### 2.17 PC12 differentiation assay

Differentiation of the PC12 cell line (kindly provided by the Neurodegeneration Laboratory at the Institut Pasteur of Montevideo, Uruguay) in response to rNGFSP was evaluated using a modified version of the protocol described by Greene and Tischler, 1979 [33]. Cells were seeded at a density of 15,000 cells per well in collagen I-coated 24-well plates, using complete RPMI medium supplemented with 10% horse serum and 10% fetal bovine serum. Following overnight attachment, the medium was replaced with RPMI containing 1% horse serum, and cells were treated with either NGF (20 ng/mL, Prospec, CYT-579) or rNGFSP (400 ng/mL). Fresh treatment medium was replenished after 48 h. Fourteen days after seeding, neuronal differentiation was assessed by quantifying the percentage of cells bearing neurites at least twice the length of the cell body.

### 2.18 Statistical analysis

All data are presented as mean ± SEM unless otherwise indicated. For immunogenicity studies, statistical comparisons of antigen-specific and cross-reactive IgG levels over time were performed using two-way analysis of variance (ANOVA) followed by Tukey’s post hoc test for multiple comparisons. Statistically significant differences relative to baseline (Day 0) are indicated in Figures. In cell-based functional assays, group comparisons were conducted using one-way ANOVA followed by Tukey’s multiple comparisons test, or unpaired two-tailed *t*-tests, depending on experimental design and number of groups. For safety evaluation in mice, hematological and biochemical parameters were summarized descriptively across timepoints (Days 0–56). In cases where apparent deviations from baseline values were observed, formal comparisons were conducted using repeated-measures ANOVA followed by Sidak’s multiple comparisons test to determine whether changes reached statistical significance. All statistical analyses were two-tailed, and a *p*-value < 0.05 was considered significant. Graphs and analyses were generated using GraphPad Prism version 10.4.2 (GraphPad Software, San Diego, CA, USA).

### 2.19 Ethical considerations

All animal studies were conducted in accordance with Uruguay’s Animal Experimentation Law No. 18.611, which aligns with the guidelines of the U.S. National Institutes of Health *Guide for the Care and Use of Laboratory Animals*. Informed consent was obtained from all dog and horse owners prior to enrollment, and no costs were incurred by the owners for their participation.

The mouse immunization protocol was reviewed and approved by the Institutional Animal Ethics Committee of the Institut Pasteur de Montevideo (Protocol #012-16, PI: LB). The rabbit immunization protocol was reviewed and approved by the Institutional Animal Ethics Committee of Xeptiva Therapeutics (Protocol #002, PI: ET).

All procedures involving dogs and horses were supervised by Dr. Nadia Crosignani (Facultad de Veterinaria, Universidad de la República, Uruguay) and Dr. María Pereira (Xeptiva Therapeutics), respectively. The dog immunization protocol was approved by the Institutional Animal Ethics Committee of Facultad de Veterinaria, Universidad de la República (Protocol #1649, PI: NC). The horse immunization protocol was reviewed and approved by the Institutional Ethical Committee for Animal Research of the Institut Pasteur de Montevideo (Protocol #017-19, PI: LB).

## 3 Results

### 3.1 Expression and purification of the recombinant antigen rNGFSP

As shown in Fig. 1A, the rNGFSP construct was cloned into the pT7 vector and designed to include an N-terminal 6 × His tag, a TEV protease cleavage site, the canine NGF domain, and a C-terminal SP sequence, that are linked by a flexible linker that codes to a GSGS peptide to improve protein folding and stability following translation. This construct was expressed recombinantly in *E. coli* BL21 (DE3) grown in Terrific Broth medium, and cells were harvested by centrifugation. The sequence alignment of the recombinant rNGFSP construct with the native canine NGF and Substance P sequences is shown in Supplementary Figure 1.

Bacterial cells were disrupted by sonication in the presence of benzonase, and inclusion bodies were isolated by centrifugation. Purified inclusion bodies were solubilized in urea, and the clarified protein solution was subjected to immobilized metal affinity chromatography. After removal of imidazole, endotoxins were eliminated using magnetic beads specifically designed for endotoxin adsorption. Finally, the protein underwent sterile filtration before formulation (Fig. 1B).

Following IMAC purification (Fig. 1C), the SDS-PAGE analysis allowed the identification of rNGFSP fusion protein as a prominent, distinct band at approximately 17.52 kDa, consistent with its calculated molecular weight (Fig. 1D).

Three independent production batches of rNGFSP were evaluated to assess the consistency of yield, purity, and endotoxin content. The production yield of rNGFSP per liter was ∼120 mg/liter of culture medium, and purity of the final product varied between 80% and 90% (Fig. 1E). Endotoxin levels remained within acceptable limits for use in pets, with all batches exhibiting values below ∼10 EU/mg of protein. Residual DNA concentrations, determined by Qubit™ fluorometric quantification, ranged from 94 to 104 × 10⁻⁶ ng/mL (Supplementary Table 1).

### 3.2 Mass spectrometry analysis of rNGFSP antigen

**Fig. 2:**
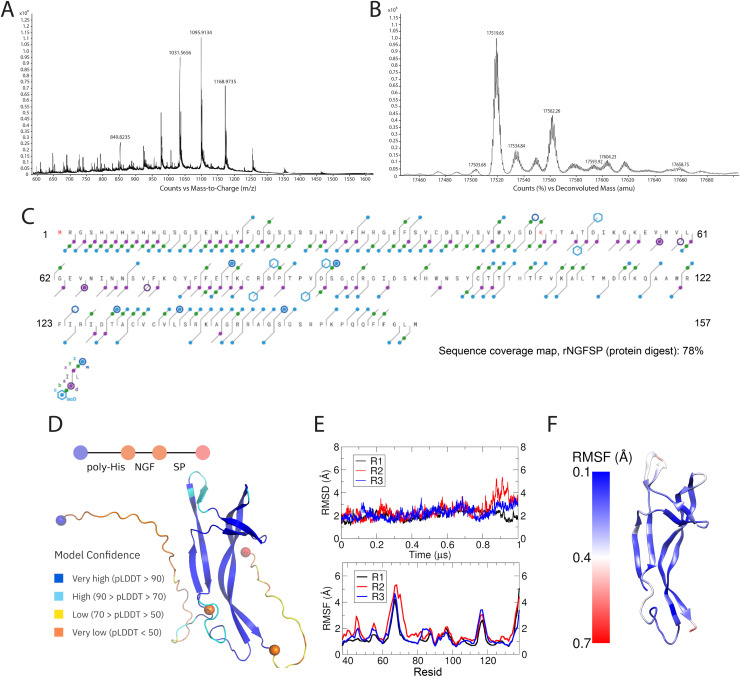
Mass spectrometry and molecular modeling analysis of NGF-SP. A) Selected part of deconvoluted MS1 spectra of NGFSP via direct injection. B) Deconvoluted MS1 spectra of NGF-SP, highlighting the main peak (red dot), the two oxidized methionine species (light blue and blue dots), and the carbamylated species (green dot). C) MS/MS Top Down analysis of NGFSP via direct injection matched to the theoretical protein sequence with two carbamyl modifications using ExDviewer. Carbamylated amino acids are indicated in red. D) Cartoon representation of one model obtained with Alphafold2. Fragment limits are marked with colored spheres and specified with a scheme at the top of the figure. Cartoons are colored according to local confidence scores. E) RMSD timelines measured over the triplicated simulations, calculated over the C-α atoms of residues 37–138, comprising the NGF region (upper panel). RMSF by residue analysis of the triplicated replicas, employing the same atom selection as in D), with secondary structure elements annotated along the sequence to facilitate identification of flexible regions (lower panel). F) RMSF values mapped onto the NGF three-dimensional structure and color-coded according to residue flexibility, providing a structural visualization of regions displaying higher conformational dynamics.

MS was used to determine the intact molecular mass and confirm the amino acid sequence of the purified recombinant rNGFSP (Fig. 2). Direct injection MS1 analysis indicated that rNGFSP exists predominantly as a monomeric peptide with a molecular mass close to the theoretical prediction of approximately 17.5 kDa (Fig. 2A and B). The presence of multiple closely spaced peaks in the mass spectrum, each differing by approximately 43 Da, suggested the occurrence of carbamoylation,a non-enzymatic post-translational modification in which carbamoyl groups (–CONH₂) are added to the amino groups of proteins. This modification results in a 43 Da increase in molecular mass per modified residue. The observed heterogeneity in rNGFSP molecular weight is consistent with partial carbamoylation, likely induced by exposure to high concentrations of urea during the solubilization and sample preparation process.

Further analysis by Top Down with ECD fragmentation (MS/MS) confirmed the predicted amino acid sequences of NGF and SP within the recombinant peptide (Fig. 2C). Sequence coverage (>75%) analysis using ExDviewer software revealed high concordance between experimentally determined and predicted sequences. Two carbamylated amino acid residues were identified (highlighted in red), confirming that observed mass heterogeneity was due to these minor chemical modifications. These results confirm both the identity and structural integrity of the recombinant antigen rNGFSP; MS/MS achieved approximately 78% sequence coverage across the rNGFSP construct, encompassing the majority of the NGF domain as well as substantial portions of the linker and SP regions. This extensive coverage confirms the presence and correct primary structure of the fusion protein over most of its length, with fragment ions matching the expected amino acid sequence with high confidence. Importantly, the detected fragments spanned both termini and internal regions of the protein, ruling out major truncations, degradation events, or rearrangements that could compromise antigen integrity. The remaining non-covered regions were consistent with known limitations of top-down MS, particularly in flexible or highly basic segments, and do not indicate loss of structural integrity.

### 3.3 *In-silico* modeling and molecular dynamics reveal structural stability of the NGF domain in rNGFSP

The predicted structure of the recombinant rNGFSP construct was obtained using AlphaFold2 modeling (Fig. 2D). A representative structural model shows clearly distinguishable domains corresponding to the poly-His tag, NGF, and SP, as indicated in the schematic (Fig. 2D). The predicted local distance difference test (pLDDT) indicates very high confidence structural prediction scores for most of the NGF region (blue), and high confidence scores (cyan) for small loops, characterized by higher mobilities. Due to their highly flexible nature, linker regions, and terminal poly-His and SP fragments, presented lower confidence scores (orange/yellow). These results were foreseeable, considering the limitations of the modelling method [34], and a dynamic characterization was further carried out using molecular simulations.

Stability of the NGF domain structure (residues 37–138) was assessed by molecular dynamics simulations in triplicate, monitoring root mean square deviation (RMSD) of C-alpha atoms over a 1 µs period (Fig. 2E). RMSD analysis revealed stable trajectories across replicates, indicating a robust and consistently folded NGF domain, with only minor conformational fluctuations.

Residue-specific flexibility was evaluated using root mean square fluctuations (RMSF) across triplicate runs (Fig. 2F). RMSF profiles identified localized flexibility primarily within loop regions, consistent among replicates. These flexible regions are visualized clearly on the structural heatmap (Fig. 2F) with higher flexibility (red) localized mainly at loop residues, contrasting with structurally rigid β-sheet domains (blue). Taken together, these in-silico analyses anticipate a stable, predominantly rigid NGF domain within the rNGFSP construct, supporting its suitability as a structurally-defined immunogen.

### 3.4 rNGFSP lacks NGF and SP biological activity in cell-based assays

To evaluate the biological activity of rNGFSP, we conducted a series of cell-based assays assessing NGF- and SP-mediated responses (Supplementary Figure 2). In the PC12 cell line, rNGFSP failed to induce neurite outgrowth, in contrast to native NGF, which promoted marked neuronal differentiation (Supplementary Figure 2A). Likewise, rNGFSP did not stimulate proliferation in TF1 cells, a well-established NGF-dependent response (Supplementary Figure 2B). In a primary mast cell degranulation assay, rNGFSP did not increase Avidin-FITC labeling, whereas SP triggered a robust degranulation response. Collectively, these results demonstrate that rNGFSP lacks the intrinsic functional NGF-like and SP-like activity in their respective cellular assays (Supplementary Figure 2C).

### 3.5 Safety and tolerance of rNGFSP in mice

To evaluate safety prior to use in other species, mice were subcutaneously immunized with high doses of rNGFSP (20 μg/dose), following the intended canine vaccination schedule (Supplementary Figure 3A). All animals completed the immunization protocol without signs of distress, behavioral alterations, or weight loss (data not shown). By Day 56, anti-rNGFSP IgG titers reached 1:16,000, confirming robust immunogenicity (Supplementary Figure 3B).

Comprehensive hematological and biochemical assessments conducted at multiple time points (Days 7 to 56) revealed no significant deviations, with all values remaining within normal physiological ranges (Supplementary Tables 2 and 3). These findings demonstrate that rNGFSP is safe and well tolerated in mice, with no evidence of systemic toxicity.

### 3.6 Immunogenicity of rNGFSP in rabbits, horses and dogs

**Fig. 3:**
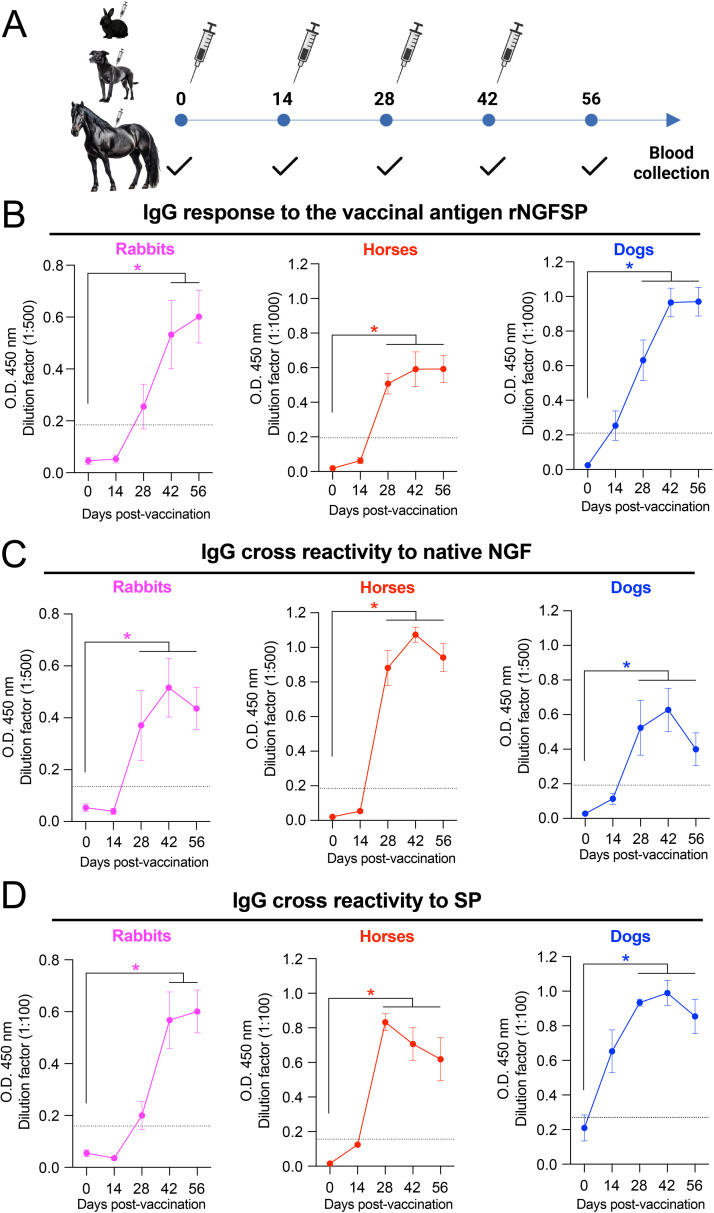
**Immunogenicity of rNGFSP in rabbits, horses, and dogs.** Rabbits (*n* = 5), horses (*n* = 3), and dogs (*n* = 6) were subcutaneously immunized with four doses of rNGFSP at two-week intervals (Days 0, 14, 28, and 42), followed by a 14-day recovery period. The formulation for dogs contained 70 µg of rNGFSP in 1 mL with 1% (v/v) Montanide™ Gel 01; horses received 700 µg in 1 mL with 5% (v/v) Montanide™ Gel 01; and rabbits received 140 µg in 2 mL with 0.5% (v/v) Montanide™ Gel 01. All formulations were prepared in 4 M urea. Serum samples were collected before the first dose (Day 0), prior to each booster dose, and on Day 56. A) Immunization schedule showing the timing of each dose and blood collection. B) Kinetics of antigen-specific IgG responses against rNGFSP measured by indirect ELISA in rabbits (magenta), horses (red), and dogs (blue). All three species exhibited a significant rise in IgG titers beginning after the second dose, with peak levels generally observed between Days 42 and 56, indicating strong immunogenicity of the vaccine formulation. C) ELISA using plates coated with recombinant native (dimeric) NGF revealed that serum IgGs from rNGFSP immunized animals bind to the endogenous form of the neurotrophin. This reactivity was evident across all species, with inter-individual variation in peak OD values but consistent induction of NGF-recognizing antibodies. D) Antibodies elicited by rNGFSP immunization also recognized SP (amidated), previously crosslinked to albumin, supporting the presence of SP-specific epitopes within the fusion immunogen. Each point represents the Mean ± SEM. *p < 0.05 compared to Day 0, determined by two-way ANOVA followed by Tukey’s multiple comparisons test.

Following vaccination with rNGFSP in rabbits, horses, and dogs (Fig. 3A) using the indicated antigen doses and adjuvant concentrations (Table 1), IgG levels against the vaccine antigen progressively increased after the first or second booster (Fig. 3B). By the end of the vaccination cycle, IgG levels against the vaccine antigen, rNGFSP, increased significantly in the three species (Fig. 3B). The anti-rNGFSP IgG levels assessed by O.D. values followed a characteristic temporal pattern, peaking after the third-fourth boosters, reaching > 32-fold increase in O.D. values, respectively, as compared with pre-immune values (Fig. 3B). Immunization of animals with the vaccine excipient failed to elicit significant levels of IgGs (data not shown).

Local reactions at the subcutaneous injection sites were monitored in rabbits, dogs, and horses throughout the immunization protocols. In rabbits, no clinically relevant local adverse reactions such as induration, edema, erythema, or ulceration were observed at any time point. In dogs, mild and transient local reactions were observed in a subset of animals. These consisted of small, well-delimited subcutaneous indurations comparable to those commonly reported after routine subcutaneous vaccinations against pathogens [35]. The size of these indurations did not exceed 2–3 cm in diameter in any case. All observed indurations resolved spontaneously and were not associated with pain on palpation, behavioral changes, lethargy, or fever. Importantly, no local reactions were detected at the time of subsequent booster administrations, two weeks after the preceding dose. All local reactions were transient and disappeared within a few days after injection.

### 3.7 rNGFSP elicits cross-reactive antibodies to native NGF and SP

Cross-reactive IgGs against NGF and SP were used as key indicators of therapeutic potential, reflecting the ability to neutralize excess endogenous peptides during inflammation (Fig. 3C and D). Rabbits, dogs, and horses vaccinated with rNGFSP showed a progressive increase in IgGs cross-reactive to the native conformations of NGF and SP, specifically recognizing dimeric NGF and C-terminally amidated SP. Antibody responses to these native forms were lower than those against rNGFSP itself and were proportional to the relative molecular size of each peptide domain. No animal showed significant anti-NGF or anti-SP IgG levels at baseline (data not shown). However, vaccinated rabbits, dogs and horses displayed a progressive increase in O.D. values after the first booster with a peak at the second or third booster.

As shown in Fig. 3C and D, rabbit sera at 1:500 and 1:100 dilutions showed increasing O.D. values against native (dimeric) NGF and amidated SP, which peaked, respectively, to a 5- and 6-fold increase in O.D. values with respect to pre-immune values after the third booster. Dog sera at 1:500 and 1:100 dilutions showed a comparable increase O.D. values against native NGF and SP, which peaked, respectively, to a 6- and 5-fold increase in O.D. values with respect to pre-immune values. In comparison, horse sera showed a higher IgG response to immunization, with a 10- and 8-fold increase in O.D. values at 1:500 and 1:100 dilutions for both native NGF and SP at D56, with respect to basal values.

### 3.8 Antibodies from dogs immunized with rNGFSP neutralize NGF and SP activities

**Fig. 4:**
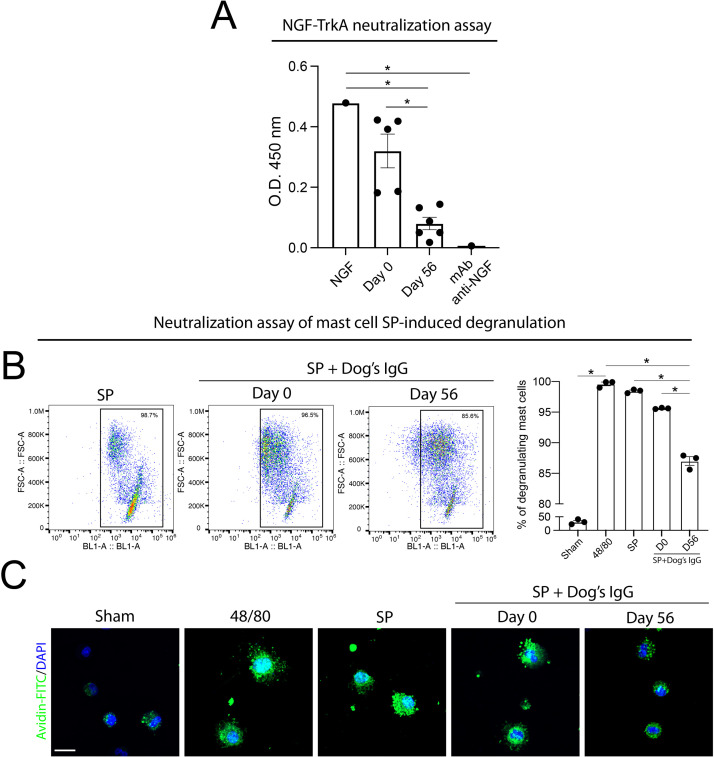
**Functional neutralization of NGF binding to the TrkA receptor and SP-induced mast cell degranulation by antibodies elicited by rNGFSP in dogs.** A) **Inhibition of NGF–TrkA binding.** Neutralization of NGF binding to the TrkA receptor was assessed by ELISA. Biotinylated NGF was incubated with TrkA in the presence of pooled sera from non-immunized dogs at day 0 (D0), sera from rNGFSP-immunized dogs at day 56 (D56), or a commercial monoclonal anti-NGF antibody (0.5 µg/mL). NGF alone served as a positive control. Sera were diluted 1:10 prior to the assay.. *p < 0.05. B) Inhibition of SP-induced mast cell degranulation. Flow cytometry analysis of mast cells stimulated with SP conjugated to BSA in the presence of IgG from non-immunized (D0) or immunized (D56) dogs. Representative plots show avidin–FITC-positive degranulating cells. The percentage of degranulating mast cells from replicate experiments is summarized on the right. All data are expressed as mean ± SEM, with *p < 0.05 compared to Day 0, determined by two-way ANOVA followed by Tukey’s multiple comparisons test. C) Confocal microscopy of mast cell degranulation. Mast cells were stained with avidin–FITC (green) and DAPI (blue). SP or the positive control compound 48/80 (10 µM) induced granule release, whereas IgG from immunized dogs (D56) reduced SP-induced degranulation. Scale bar: 10 µm.

To evaluate the functional activity of antibodies elicited by rNGFSP immunization, we first assessed their capacity to neutralize NGF binding to the TrkA receptor. In an ELISA-based assay, pooled sera from dogs collected after rNGFSP immunization (Day 56) significantly reduced NGF binding to TrkA, as compared to NGF alone and the same sera obtained from dogs before immunization (Day 0) (Fig. 4A). In comparison, commercial monoclonal anti-NGF antibody induced complete inhibition.

We next investigated whether these antibodies could inhibit mast cell degranulation induced by SP. Flow cytometry analysis showed that SP conjugated to albumin triggered robust degranulation in mast cells, as indicated by a high percentage of avidin–FITC-positive cells (Fig. 4B). The presence of IgG from non-immunized dogs (Day 0) did not alter this response. However, IgG purified from Day 56 sera of immunized dogs significantly reduced SP-induced mast cell degranulation. Confocal microscopy confirmed these findings. SP and the positive degranulation control compound 48/80 induced marked granule release, visualized by avidin–FITC staining (Fig. 4C). In contrast, SP stimulation in the presence of IgG from immunized dogs (Day 56) resulted in fewer avidin–positive granules, consistent with inhibition of mast cell activation. Together, these results indicate that antibodies induced by rNGFSP immunization can neutralize NGF–TrkA binding and suppress SP-induced mast cell degranulation.

## 4 Discussion

This study reports the development and characterization of rNGFSP, a novel recombinant fusion protein engineered as an immunogen to elicit therapeutic antibodies against NGF and SP, two neuropeptides that act synergistically to promote inflammation and pain associated with degenerative joint disease [[36], [37], [38]]. The rNGFSP construct integrates NGF and SP sequences into a structurally non-native scaffold lacking intrinsic biological activity, while preserving the ability to induce an immune response. Immunization with rNGFSP formulated as a vaccine, resulted in the generation of antibodies that recognized the native conformations of both NGF and SP and inhibited their respective biological activities in vitro, indicating the functional relevance of the humoral response. These findings support the potential of rNGFSP as a therapeutic vaccine targeting critical components of the neuroimmune axis implicated in OA pathophysiology. rNGFSP-based vaccination may provide broader modulation of pain and inflammation pathways, and enhanced therapeutic efficacy compared to existing monoclonal antibody therapies that selectively neutralize NGF [17,39].

The production of recombinant immunogens in *E. coli* followed by recovery from inclusion bodies is a well-established approach in biotechnology [40,41], particularly for antigens intended for use in veterinary vaccines. Consistent with this methodology, our expression system yielded high levels of the rNGFSP fusion protein, which accumulated predominantly in inclusion bodies. These aggregates were efficiently solubilized using 8 M urea, allowing recovery of the target protein under denaturing conditions. Purification of rNGFSP was conducted via IMAC based on the N-terminal poly-histidine tag contained in the protein. This process resulted in high protein recovery with purity levels in the range of 80–90%, and with endotoxin concentrations sufficiently low to permit use in experimental immunogenic formulations. The workflow employed is consistent with established industry standards for the scalable and cost-effective production of recombinant proteins and subunit vaccines [42], offering practical advantages for translational applications in veterinary settings. Although proteins recovered from denaturing conditions typically lack native folding, our results indicate that rNGFSP retains key structural elements necessary for immunogenicity. These findings demonstrate that rNGFSP can be produced at a quality appropriate for further development as a therapeutic immunogen. This is especially relevant in veterinary medicine, where there is a growing demand for economical and effective immunization strategies to manage chronic inflammatory and painful conditions such as OA.

Mass spectrometry analysis confirmed that the recombinant rNGFSP protein had the amino acid sequence integrity and expected molecular mass consistent with its amino acid sequence. The high MS/MS sequence coverage, together with intact mass determination and the absence of unexpected fragment patterns, supports that the purified rNGFSP corresponds to a full-length, structurally intact recombinant protein suitable for downstream immunological applications. Some molecular species showed mass shifts attributed to carbamoylation, which may be related to urea exposure during antigen solubilization [43]. We have not determined whether carbamoylation could introduce additional antigenic determinants within the immunogen. However, since carbamoylated species were low relative to the overall protein, the limited extent of this modification suggests that carbamoylation is unlikely to represent a dominant driver of the immune response elicited by rNGFSP [43]. Further studies comparing alternative formulations or directly assessing antibody reactivity against carbamoylated versus non-carbamoylated antigen preparations will be required to formally define the contribution of such modifications to antigenicity .

Structural modeling using AlphaFold predicted the three-dimensional conformation of rNGFSP, revealing that even under non-physiological folding conditions, the protein contains a stable central core structurally comparable to mature NGF [44], while the carboxy-terminal SP sequence remains highly flexible and solvent-exposed. Based on its structural properties, rNGFSP is expected to function as an engineered neoantigen-like immunogen not naturally present in the host, capable of breaking immune tolerance and eliciting antibodies cross-reactive with endogenous NGF and SP. rNGFSP lacks the native conformation and post-translational modifications of its parental peptides, including the dimeric structure of NGF and the C-terminal amidation of SP. This absence of native folding is consistent with the mechanism described for other self-antigen vaccines [45]. Consequently, rNGFSP appears to effectively bypass natural tolerance mechanisms that usually prevent antibody responses to self-peptides.

Murine models have been previously validated for assessing the safety and potency of recombinant immunogens [46]. Consistent with this, biological safety analyses conducted in mice following active immunization with rNGFSP at relatively high doses, formulated with 1% Montanide Gel 01 adjuvant [47], demonstrated good tolerability throughout the vaccination protocol. No systemic adverse effects were observed, and hematological and biochemical parameters remained within normal ranges, indicating a favorable safety profile. These results support the suitability of rNGFSP for further therapeutic development and translational application in other species.

Vaccination with rNGFSP elicited a robust antibody response in rabbits, dogs, and horses, even when formulated with relatively low concentrations (0.5–5%) of Montanide Gel 01 adjuvant—substantially below the optimal concentration reported in previous studies [48]. This finding further supports the strong immunogenicity of rNGFSP. Importantly, the elicited antibodies were cross-reactive with the physiological conformations of both NGF and SP, indicating that rNGFSP contains epitopes structurally related to those present in the native peptides. These results suggest that the recombinant immunogen effectively mimics key antigenic features of the target molecules despite its non-native folding. The rNGFSP doses employed were exploratory and informed by dosing ranges used in prior studies of self-antigen vaccines in canine, porcine, and equine models [[49], [50], [51]]. Across all tested species, the vaccine was well tolerated, with only mild and transient local reactions at the injection site, supporting its potential for further development as a therapeutic vaccine for use in veterinary medicine.

Cell culture experiments demonstrated that rNGFSP lacks the intrinsic biological activity of its parental molecules, NGF and SP. This finding aligns with previous studies showing that NGF requires a specific homodimer conformation for TrkA receptor activation [52], and that SP activity depends on C-terminal amidation [53]. The absence of NGF- or SP-like activity in rNGFSP is a critical feature for its intended use as a therapeutic vaccine, ensuring that subcutaneous administration does not induce undesired physiological effects mediated by either neuropeptide such as hyperalgesia or neurogenic inflammation [[54], [55], [56], [57], [58]].

Functional in vitro assays demonstrated that IgGs elicited by rNGFSP immunization in dogs effectively neutralized specific biological activities mediated by NGF and SP. Using pooled sera or purified IgGs from vaccinated animals, we observed a significant reduction in NGF binding to the extracellular domain of the TrkA receptor, as well as inhibition of NGF-induced proliferation of TF-1 cells, compared to preimmune controls. These assays are well-established and have been previously validated for detecting NGF inhibitors, including monoclonal antibodies [30].

In addition, IgGs from vaccinated dogs significantly inhibited SP-induced degranulation of mast cells differentiated from murine bone marrow, as described by Gaudenzio *et al*. [59]. While earlier studies have reported protective effects of passive immunization with polyclonal anti-SP antibodies in vivo [60,61], our results represent the first direct evidence of SP neutralization by specific antibodies in a cell-based assay. These findings indicate the functional relevance of the humoral response elicited by rNGFSP and its capacity to interfere with key effector pathways involved in neurogenic inflammation. Mast cells play a central role in SP-mediated nociception and inflammation in joint diseases, as upon activation, they release granules containing histamine, serotonin, proteases, and trophic factors such as NGF, which contribute to pain sensitization and joint damage [36,62,63]. The observed inhibition of NGF- and SP-induced activities indicates the potential of rNGFSP to modulate critical neuroimmune mechanisms implicated in OA pathogenesis.

Vaccines directed against self-antigens have been previously developed in both veterinary and human medicine; for example, GnRF-based vaccines for immunocastration are approved for use in pigs and dogs [51,64], and human clinical trials have evaluated vaccines targeting angiotensin and chorionic gonadotropin with acceptable safety profiles [65,66]. Anti-NGF monoclonal antibodies have been developed for the treatment of OA pain in humans, although none have received regulatory approval due to safety concerns [67]. In veterinary medicine, however, the anti-NGF antibodies bedinvetmab and frunevetmab have been approved for managing OA-related pain in dogs and cats, respectively, showing consistent efficacy and tolerability [16,68,69]. While these advances further support the therapeutic potential of NGF blockade in OA, active immunization strategies remain underexplored in clinical trials, despite the promising results of an experimental immunogen composed of NGF-derived peptides conjugated to virus-like particles in preclinical OA models [21,22].

In contrast, no antibody-based OA therapies have been developed against SP, despite strong experimental evidence supporting its role in neurogenic inflammation and nociception, and the therapeutic potential of targeting SP receptors in musculoskeletal diseases [38,70,71]. SP promotes joint inflammation and pain primarily via NK1R and MRGPR-mediated activation of mast cells, leading to the release of inflammatory mediators including NGF [4,72,73]. In this context, rNGFSP represents a novel dual-target immunogen capable of modulating two key neuroimmune mediators, offering a potential therapeutic advantage in the management of chronic inflammatory joint diseases such as OA.

Collectively, these findings support rNGFSP as a promising candidate for active immunization against chronic inflammatory pain in OA. By simultaneously targeting NGF and SP, rNGFSP offers a multimodal mechanism of action that addresses key limitations of current therapies, including the limited scope of NSAIDs and the high cost and dosing constraints of monoclonal antibody treatments. Long-term management of canine degenerative joint disease presents significant clinical challenges, such as poor compliance with daily oral NSAID administration and related adverse side effects [74,75]. While anti-NGF monoclonal antibodies offer an alternative via monthly subcutaneous injections, recent safety concerns and limited affordability in low-income settings suggest the need for more accessible solutions [76]. Active immunization with rNGFSP may overcome these limitations by inducing a durable antibody response that neutralizes excess NGF and SP without disrupting their basal physiological roles. This strategy holds potential not only for sustained pain control and reduced inflammation but also for improved treatment adherence and cost-effectiveness. Further studies are warranted to evaluate the efficacy, safety, and duration of the therapeutic response in canine degenerative joint disease.

## Declaration of generative AI and AI-assisted technologies in the writing process

During the preparation of this work, the authors used generative AI tools solely to assist in improving the clarity and readability of the manuscript. The authors reviewed and edited all content as needed and take full responsibility for the content of the published article.

## CRediT authorship contribution statement

**Valentina Varela:** Writing – review & editing, Writing – original draft, Validation, Supervision, Methodology, Investigation, Formal analysis, Data curation, Conceptualization. **Monique Costa:** Writing – review & editing, Methodology, Investigation, Formal analysis, Data curation. **Cecilia Maciel:** Methodology, Investigation, Formal analysis, Data curation. **Joaquín Barbeito:** Methodology, Investigation, Formal analysis, Data curation. **Exequiel E. Barrera:** Writing – review & editing, Writing – original draft, Methodology, Investigation, Formal analysis, Data curation. **Erica Gutierre:** Methodology, Investigation. **Agustín Correa:** Methodology, Investigation, Formal analysis. **Melania Elgue:** Methodology, Investigation. **Sebastián Carrasco:** Methodology, Investigation. **Magdalena Domínguez Larrosa:** Methodology, Investigation. **María Pereira:** Methodology, Investigation. **Josefina Correa:** Writing – review & editing, Supervision, Funding acquisition. **Nadia Crosignani:** Supervision, Project administration, Methodology. **Joseph S. Beckman:** Writing – review & editing, Funding acquisition, Conceptualization. **Luis Barbeito:** Writing – review & editing, Writing – original draft, Validation, Supervision, Project administration, Formal analysis, Data curation, Conceptualization. **Emiliano Trias:** Writing – review & editing, Writing – original draft, Visualization, Validation, Supervision, Project administration, Methodology, Funding acquisition, Formal analysis, Data curation, Conceptualization.

## Declaration of competing interest

Valentina Varela, Josefina Correa, Luis Barbeito, and Emiliano Trias are co-founders and shareholders of Xeptiva Therapeutics.

Valentina Varela, Luis Barbeito, and Emiliano Trias are inventors of one patent related to the technology described herein. International Patent Application No. PCT/IB2023/050517.

Valentina Varela, Monique Costa, Cecilia Maciel, Melania Elgue, Magdalena Domínguez, María Pereira, and Luis Barbeito are or have been employees of, or have received compensation from, Xeptiva Therapeutics at some point during the conduct of the studies presented in this work.

Agustín Correa was employed by the Institut Pasteur de Montevideo during the course of this study. He is currently a co-founder of the biotechnology startup Scaffold Biotech.

Nadia Crosignani, Erica Gutierre, and Sebastián Carrasco are employees of the Facultad de Veterinaria, Universidad de la República.

Joseph Beckman is a shareholder of Xeptiva Therapeutics.

The work presented here was funded by Xeptiva Therapeutics, which also received grants from the National Agency for Research and Innovation (ANII) of Uruguay.

## Data Availability

All data generated or analyzed during this study are included in this published article and its supplementary information files.

## Appendix Supplementary materials

Image, application 1
