# Supplementary material for: Production and characterization of rNGFSP: a recombinant fusion immunogen eliciting dual anti-NGF and anti-Substance P therapeutic antibodies for Degenerative Joint Disease
Source: Biotechnol Rep (Amst). 2026 Jan 12;49:e00946. doi: 10.1016/j.btre.2026.e00946 (PMC12834844; doi:10.1016/j.btre.2026.e00946)
Supplement: Supplementary file 1 [file mmc1.docx]

**Supplementary Material**

**Supplementary Materials and Methods**

**pT7 plasmid sequence encoding rNGFSP**

TGGCGAATGGGACGCGCCCTGTAGCGGCGCATTAAGCGCGGCGGGTGTGGTGGTTACGCGCAGCGTGACCGCTACACTTGCCAGCGCCCTAGCGCCCGCTCCTTTCGCTTTCTTCCCTTCCTTTCTCGCCACGTTCGCCGGCTTTCCCCGTCAAGCTCTAAATCGGGGGCTCCCTTTAGGGTTCCGATTTAGTGCTTTACGGCACCTCGACCCCAAAAAACTTGATTAGGGTGATGGTTCACGTAGTGGGCCATCGCCCTGATAGACGGTTTTTCGCCCTTTGACGTTGGAGTCCACGTTCTTTAATAGTGGACTCTTGTTCCAAACTGGAACAACACTCAACCCTATCTCGGTCTATTCTTTTGATTTATAAGGGATTTTGCCGATTTCGGCCTATTGGTTAAAAAATGAGCTGATTTAACAAAAATTTAACGCGAATTTTAACAAAATATTAACGTTTACAATTTCAGGTGGCACTTTTCGGGGAAATGTGCGCGGAACCCCTATTTGTTTATTTTTCTAAATACATTCAAATATGTATCCGCTCATGAGACAATAACCCTGATAAATGCTTCAATAATATTGAAAAAGGAAGAGTATGAGTATTCAACATTTCCGTGTCGCCCTTATTCCCTTTTTTGCGGCATTTTGCCTTCCTGTTTTTGCTCACCCAGAAACGCTGGTGAAAGTAAAAGATGCTGAAGATCAGTTGGGTGCACGAGTGGGTTACATCGAACTGGATCTCAACAGCGGTAAGATCCTTGAGAGTTTTCGCCCCGAAGAACGTTTTCCAATGATGAGCACTTTTAAAGTTCTGCTATGTGGCGCGGTATTATCCCGTATTGACGCCGGGCAAGAGCAACTCGGTCGCCGCATACACTATTCTCAGAATGACTTGGTTGAGTACTCACCAGTCACAGAAAAGCATCTTACGGATGGCATGACAGTAAGAGAATTATGCAGTGCTGCCATAACCATGAGTGATAACACTGCGGCCAACTTACTTCTGACAACGATCGGAGGACCGAAGGAGCTAACCGCTTTTTTGCACAACATGGGGGATCATGTAACTCGCCTTGATCGTTGGGAACCGGAGCTGAATGAAGCCATACCAAACGACGAGCGTGACACCACGATGCCTGCAGCAATGGCAACAACGTTGCGCAAACTATTAACTGGCGAACTACTTACTCTAGCTTCCCGGCAACAATTAATAGACTGGATGGAGGCGGATAAAGTTGCAGGACCACTTCTGCGCTCGGCCCTTCCGGCTGGCTGGTTTATTGCTGATAAATCTGGAGCCGGTGAGCGTGGGTCTCGCGGTATCATTGCAGCACTGGGGCCAGATGGTAAGCCCTCCCGTATCGTAGTTATCTACACGACGGGGAGTCAGGCAACTATGGATGAACGAAATAGACAGATCGCTGAGATAGGTGCCTCACTGATTAAGCATTGGTAACTGTCAGACCAAGTTTACTCATATATACTTTAGATTGATTTAAAACTTCATTTTTAATTTAAAAGGATCTAGGTGAAGATCCTTTTTGATAATCTCATGACCAAAATCCCTTAACGTGAGTTTTCGTTCCACTGAGCGTCAGACCCCGTAGAAAAGATCAAAGGATCTTCTTGAGATCCTTTTTTTCTGCGCGTAATCTGCTGCTTGCAAACAAAAAAACCACCGCTACCAGCGGTGGTTTGTTTGCCGGATCAAGAGCTACCAACTCTTTTTCCGAAGGTAACTGGCTTCAGCAGAGCGCAGATACCAAATACTGTCCTTCTAGTGTAGCCGTAGTTAGGCCACCACTTCAAGAACTCTGTAGCACCGCCTACATACCTCGCTCTGCTAATCCTGTTACCAGTGGCTGCTGCCAGTGGCGATAAGTCGTGTCTTACCGGGTTGGACTCAAGACGATAGTTACCGGATAAGGCGCAGCGGTCGGGCTGAACGGGGGGTTCGTGCACACAGCCCAGCTTGGAGCGAACGACCTACACCGAACTGAGATACCTACAGCGTGAGCTATGAGAAAGCGCCACGCTTCCCGAAGGGAGAAAGGCGGACAGGTATCCGGTAAGCGGCAGGGTCGGAACAGGAGAGCGCACGAGGGAGCTTCCAGGGGGAAACGCCTGGTATCTTTATAGTCCTGTCGGGTTTCGCCACCTCTGACTTGAGCGTCGATTTTTGTGATGCTCGTCAGGGGGGCGGAGCCTATGGAAAAACGCCAGCAACGCGGCCTTTTTACGGTTCCTGGCCTTTTGCTGGCCTTTTGCTCACATGTTCTTTCCTGCGTTATCCCCTGATTCTGTGGATAACCGTATTACCGCCTTTGAGTGAGCTGATACCGCTCGCCGCAGCCGAACGACCGAGCGCAGCGAGTCAGTGAGCGAGGAAGCGGAAGAGCGCCTGATGCGGTATTTTCTCCTTACGCATCTGTGCGGTATTTCACACCGCATATATGGTGCACTCTCAGTACAATCTGCTCTGATGCCGCATAGTTAAGCCAGTATACACTCCGCTATCGCTACGTGACTGGGTCATGGCTGCGCCCCGACACCCGCCAACACCCGCTGACGCGCCCTGACGGGCTTGTCTGCTCCCGGCATCCGCTTACAGACAAGCTGTGACCGTCTCCGGGAGCTGCATGTGTCAGAGGTTTTCACCGTCATCACCGAAACGCGCGAGGCAGCTGCGGTAAAGCTCATCAGCGTGGTCGTGAAGCGATTCACAGATGTCTGCCTGTTCATCCGCGTCCAGCTCGTTGAGTTTCTCCAGAAGCGTTAATGTCTGGCTTCTGATAAAGCGGGCCATGTTAAGGGCGGTTTTTTCCTGTTTGGTCACTGATGCCTCCGTGTAAGGGGGATTTCTGTTCATGGGGGTAATGATACCGATGAAACGAGAGAGGATGCTCACGATACGGGTTACTGATGATGAACATGCCCGGTTACTGGAACGTTGTGAGGGTAAACAACTGGCGGTATGGATGCGGCGGGACCAGAGAAAAATCACTCAGGGTCAATGCCAGCGCTTCGTTAATACAGATGTAGGTGTTCCACAGGGTAGCCAGCAGCATCCTGCGATGCAGATCCGGAACATAATGGTGCAGGGCGCTGACTTCCGCGTTTCCAGACTTTACGAAACACGGAAACCGAAGACCATTCATGTTGTTGCTCAGGTCGCAGACGTTTTGCAGCAGCAGTCGCTTCACGTTCGCTCGCGTATCGGTGATTCATTCTGCTAACCAGTAAGGCAACCCCGCCAGCCTAGCCGGGTCCTCAACGACAGGAGCACGATCATGCGCACCCGTGGGGCCGCCATGCCGGCGATAATGGCCTGCTTCTCGCCGAAACGTTTGGTGGCGGGACCAGTGACGAAGGCTTGAGCGAGGGCGTGCAAGATTCCGAATACCGCAAGCGACAGGCCGATCATCGTCGCGCTCCAGCGAAAGCGGTCCTCGCCGAAAATGACCCAGAGCGCTGCCGGCACCTGTCCTACGAGTTGCATGATAAAGAAGACAGTCATAAGTGCGGCGACGATAGTCATGCCCCGCGCCCACCGGAAGGAGCTGACTGGGTTGAAGGCTCTCAAGGGCATCGGTCGAGATCCCGGTGCCTAATGAGTGAGCTAACTTACATTAATTGCGTTGCGCTCACTGCCCGCTTTCCAGTCGGGAAACCTGTCGTGCCAGCTGCATTAATGAATCGGCCAACGCGCGGGGAGAGGCGGTTTGCGTATTGGGCGCCAGGGTGGTTTTTCTTTTCACCAGTGAGACGGGCAACAGCTGATTGCCCTTCACCGCCTGGCCCTGAGAGAGTTGCAGCAAGCGGTCCACGCTGGTTTGCCCCAGCAGGCGAAAATCCTGTTTGATGGTGGTTAACGGCGGGATATAACATGAGCTGTCTTCGGTATCGTCGTATCCCACTACCGAGATGTCCGCACCAACGCGCAGCCCGGACTCGGTAATGGCGCGCATTGCGCCCAGCGCCATCTGATCGTTGGCAACCAGCATCGCAGTGGGAACGATGCCCTCATTCAGCATTTGCATGGTTTGTTGAAAACCGGACATGGCACTCCAGTCGCCTTCCCGTTCCGCTATCGGCTGAATTTGATTGCGAGTGAGATATTTATGCCAGCCAGCCAGACGCAGACGCGCCGAGACAGAACTTAATGGGCCCGCTAACAGCGCGATTTGCTGGTGACCCAATGCGACCAGATGCTCCACGCCCAGTCGCGTACCGTCTTCATGGGAGAAAATAATACTGTTGATGGGTGTCTGGTCAGAGACATCAAGAAATAACGCCGGAACATTAGTGCAGGCAGCTTCCACAGCAATGGCATCCTGGTCATCCAGCGGATAGTTAATGATCAGCCCACTGACGCGTTGCGCGAGAAGATTGTGCACCGCCGCTTTACAGGCTTCGACGCCGCTTCGTTCTACCATCGACACCACCACGCTGGCACCCAGTTGATCGGCGCGAGATTTAATCGCCGCGACAATTTGCGACGGCGCGTGCAGGGCCAGACTGGAGGTGGCAACGCCAATCAGCAACGACTGTTTGCCCGCCAGTTGTTGTGCCACGCGGTTGGGAATGTAATTCAGCTCCGCCATCGCCGCTTCCACTTTTTCCCGCGTTTTCGCAGAAACGTGGCTGGCCTGGTTCACCACGCGGGAAACGGTCTGATAAGAGACACCGGCATACTCTGCGACATCGTATAACGTTACTGGTTTCACATTCACCACCCTGAATTGACTCTCTTCCGGGCGCTATCATGCCATACCGCGAAAGGTTTTGCGCCATTCGATGGTGTCCGGGATCTCGACGCTCTCCCTTATGCGACTCCTGCATTAGGAAGCAGCCCAGTAGTAGGTTGAGGCCGTTGAGCACCGCCGCCGCAAGGAATGGTGCATGCAAGGAGATGGCGCCCAACAGTCCCCCGGCCACGGGGCCTGCCACCATACCCACGCCGAAACAAGCGCTCATGAGCCCGAAGTGGCGAGCCCGATCTTCCCCATCGGTGATGTCGGCGATATAGGCGCCAGCAACCGCACCTGTGGCGCCGGTGATGCCGGCCACGATGCGTCCGGCGTAGAGGATCGAGATCGATCTCGATCCCGCGAAATTAATACGACTCACTATAGGGGAATTGTGAGCGGATAACAATTCCCCTCTAGAAATAATTTTGTTTAACTTTAAGAAGGAGATATACATATGAGAGGATCGCATCACCATCACCATCACGGTAGCGGATCGGAAAACCTGTATTTTCAGGGATCCTCTTCTTCTCACCCGGTTTTCCACCGTGGTGAATTCTCTGTTTGCGACTCTGTTTCTGTTTGGGTTGGTGACAAAACCACCGCTACCGACATCAAAGGTAAAGAAGTTATGGTTCTGGGTGAAGTTAACATCAACAACTCTGTTTTCAAACAGTACTTCTTCGAAACCAAATGCCGTGACCCGACCCCGGTTGACTCTGGTTGCCGTGGTATCGACTCTAAACACTGGAACTCTTACTGCACCACCACCCACACCTTCGTTAAAGCTCTGACCATGGACGGTAAACAGGCTGCGTGGAGGTTCATCCGTATCGACACCGCTTGCGTTTGCGTTCTGTCTCGTAAAGCTGGTCGTCGTGCTGGTTCTGGTTCTCGTCCGAAACCGCAGCAGTTCTTCGGTCTGATGTAACTCGAGCACCACCACCACCACCACTGAGATCCGGCTGCTAACAAAGCCCGAAAGGAAGCTGAGTTGGCTGCTGCCACCGCTGAGCAATAACTAGCATAACCCCTTGGGGCCTCTAAACGGGTCTTGAGGGGTTTTTTGCTGAAAGGAGGAACTATATCCGGAT

**Supplementary Figure 1. Schematic representation and sequence alignment of the rNGFSP fusion antigen.** The full amino acid sequence of the recombinant rNGFSP antigen is shown, highlighting its modular design. The N-terminal region includes a 6×His tag (green) followed by a flexible linker and a TEV protease cleavage site (cyan). The NGF region derived from *Canis lupus* is shown in red, corresponding to the mature NGF sequence. A second linker (magenta) connects NGF to the SP region (blue), also derived from *Canis lupus*. Below, the individual sequences of mature canine NGF and SP are displayed for comparison, illustrating their alignment within the recombinant fusion construct.

**Supplementary Figure 2. rNGFSP lacks NGF- and SP-like biological activity in cell-based functional assays.** A) Effect of rNGFSP on NGF-dependent PC12 cell differentiation assay. PC12 cells were cultured on collagen I-coated plates and treated with vehicle (Untreated), NGF (20 ng/mL), or rNGFSP (400 ng/mL) for 14 days. Native NGF induced marked neurite outgrowth, as evidenced by elongated projections characteristic of neuronal differentiation (middle panel), while rNGFSP-treated cells showed no morphological changes and were indistinguishable from untreated controls (left and right panels). Differentiated cells—defined as those bearing neurites at least twice the length of the soma—were quantified from three independent experiments. Representative images and quantification from three independent experiments are shown. The graph quantifies the number of differentiated cells per field, showing a significant increase in the NGF group (*p < 0.001). B) Effect of rNGFSP on NGF-dependent TF1 cell proliferation assay. TF1 cells were cultured in RPMI containing 5% FBS and treated with NGF (20 ng/mL) or rNGFSP (400 ng/mL). Cell proliferation was measured after 72 hours using CCK-8 reagent and expressed as arbitrary units of absorbance at 460 nm. NGF induced significant proliferation, while rNGFSP did not differ from the untreated group. The graph shows a significant increase in cell number only in the NGF-treated group (*p < 0.001). C) Substance P-dependent mast cell degranulation assay. Bone marrow-derived murine mast cells were treated for 30 minutes with SP (10 µM), rNGFSP (10 µM), or vehicle control. Mast cell degranulation was assessed by flow cytometry using Avidin-FITC labeling of mast cell extracellular granule-associated contents. SP induced a marked increase in Avidin-labelled cells denoting degranulation, while rNGFSP failed to increase Avidin-FITC labeling above baseline levels. Quantification shown as mean ± SD; *p < 0.05 by one-way ANOVA with Tukey’s post hoc test.

**Supplementary Figure 3. Immunogenicity of the rNGFSP vaccine in mice.** A) Schematic representation of the immunization schedule used in C57BL/6 mice. Animals received 120 µg of rNGFSP formulated with 1% Montanide™ Gel 01 in a final volume of 200 µL via subcutaneous injection on Days 0, 14, 28, and 42. Blood was collected on Day 56 for antibody analysis. B) Evaluation of the humoral immune response. Anti-rNGFSP IgG titers measured by endpoint dilution ELISA at Day 0 and Day 56. Data are shown as individual values with mean ± SEM. Statistical significance was determined using a paired t-test; *p < 0.05.

**Supplementary Table 1. Purity parameters of formulated vaccines.** Quality attributes of the formulated product batches, showing protein purity (%), endotoxin levels (EU/mg), residual DNA content (ng/mL), and percentage of contaminant proteins.

**Supplementary Table 2. Hematological parameters in mice pre-immunization and at Day 56 after rNGFSP administration.** Complete blood count results for control and rNGFSP-treated mice. Values represent mean ± SD and range (Min–Max) for each group. Blood samples were collected before the first immunization (Preimmune) and at Day 56. No clinically significant differences were observed between groups. Additional intermediate timepoints (Days 7, 21, 35, and 49) are provided in the Supplementary Material.

**Supplementary Table 3. Serum biochemical parameters in mice pre-immunization and at Day 56 after rNGFSP administration.** Representative liver and kidney function markers in control and rNGFSP-treated mice. Data show mean ± SD and range (Min–Max) for each group, at baseline (Preimmune) and Day 56. No abnormalities or significant alterations outside the physiological range were detected. Full biochemical profiles across all timepoints are available in the Supplementary Material.

**Supplementary Figure 1**

**
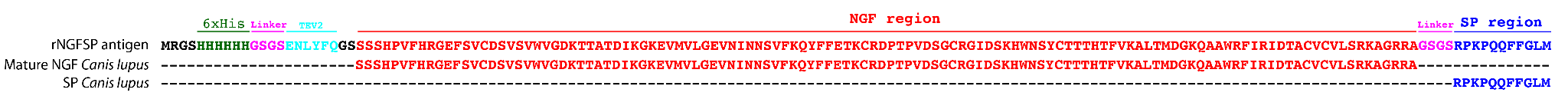
**

**Supplementary Figure 2**


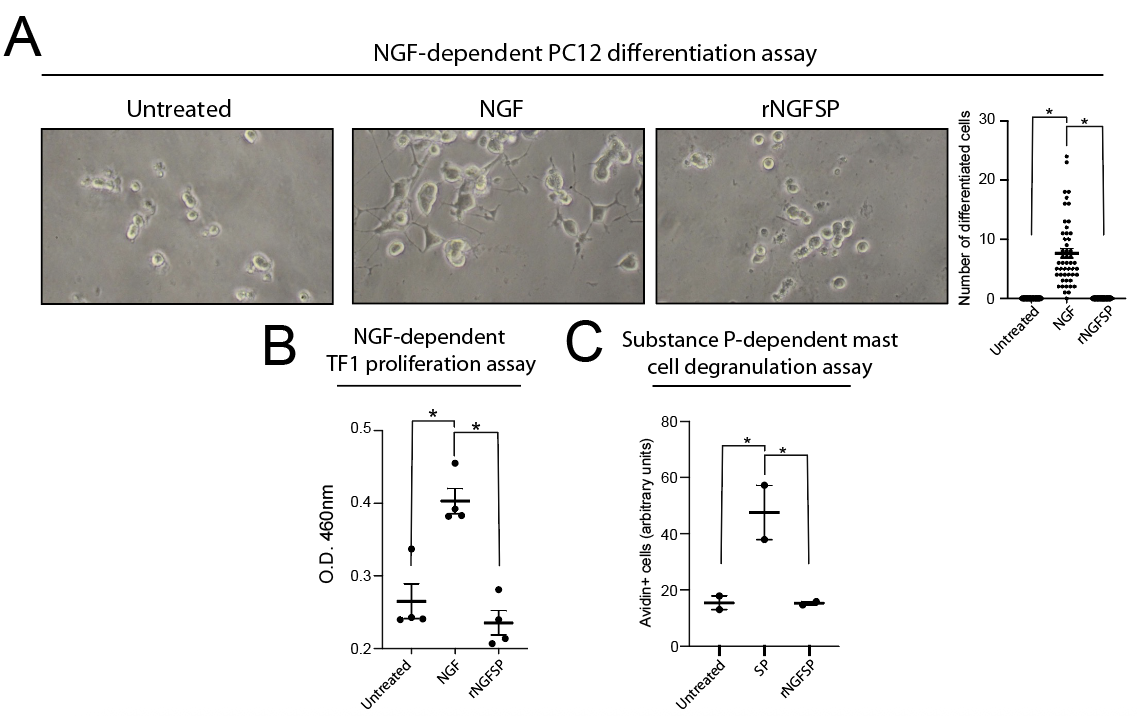


**Supplementary Figure 3**


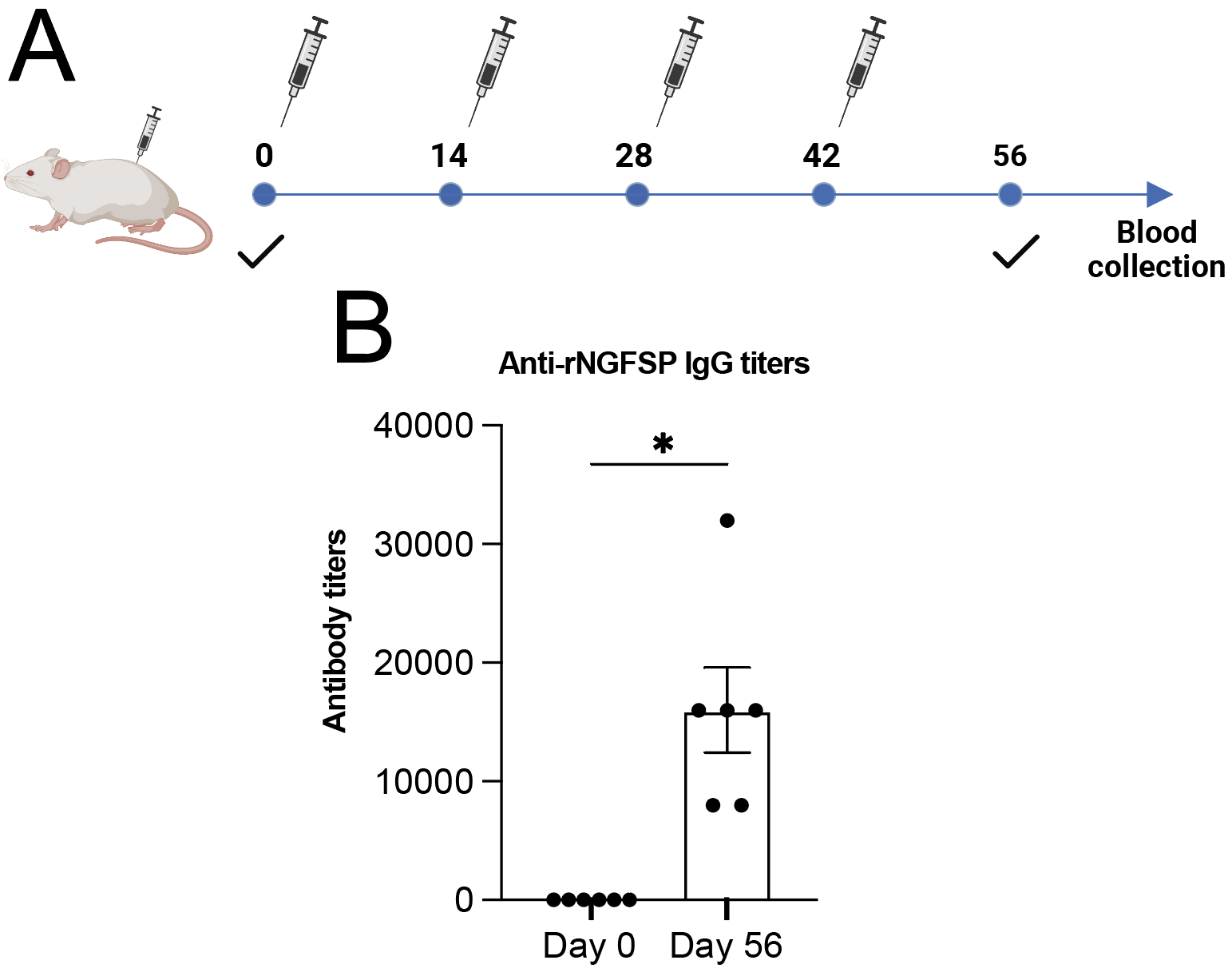


**Supplementary Table 1**

|  | Parameters | | | |
| --- | --- | --- | --- | --- |
|  | **% purity** | **Endotoxin (EU/mg)** | **DNA content (ng/mL)** | **% contaminant proteins** |
| Batch 1 | ≃80 | < 3 | 94.8 x 10^-6^ | ≃20 |
| Batch 2 | ≃85 | < 3 | 96.2 x 10^-6^ | **≃**15 |
| Batch 3 | ≃82 | < 5 | 104 x 10^-6^ | ≃18 |

**Supplementary Table 2**

| **Parameter** | **Day** | **Excipient** | | | | | | **rNGFSP** | | | | | | p-  value |
| --- | --- | --- | --- | --- | --- | --- | --- | --- | --- | --- | --- | --- | --- | --- |
|  |  | Mean ± SD | | | MIN - MAX | | | Mean ± SD | | | MIN - MAX | | |  |
| White blood cell count 10^9/L | D0 | 9.31 | ± | 0.18 | 9.20 | - | 9.52 | 11.51 | ± | 3.78 | 7.30 | - | 14.62 | 0.83 |
|  | D56 | 7.43 | ± | 3.96 | 4.14 | - | 11.82 | 8.00 | ± | 1.29 | 6.66 | - | 9.23 |  |
| Neutrophils 10^9/L | D0 | 0.39 | ± | 0.11 | 0.27 | - | 0.48 | 1.70 | ± | 2.38 | 0.30 | - | 4.44 | 0.87 |
|  | D56 | 0.42 | ± | 0.22 | 0.20 | - | 0.64 | 0.46 | ± | 0.33 | 0.13 | - | 0.78 |  |
| Lymphocytes 10^9/L | D0 | 8.43 | ± | 0.33 | 8.07 | - | 8.73 | 9.03 | ± | 2.48 | 6.68 | - | 11.63 | 0.76 |
|  | D56 | 6.53 | ± | 3.20 | 3.79 | - | 10.05 | 7.17 | ± | 0.76 | 6.44 | - | 7.96 |  |
| Monocytes 10^9/L | D0 | 0.28 | ± | 0.13 | 0.20 | - | 0.43 | 0.47 | ± | 0.44 | 0.15 | - | 0.97 | 0.81 |
|  | D56 | 0.24 | ± | 0.31 | 0.03 | - | 0.59 | 0.19 | ± | 0.13 | 0.04 | - | 0.28 |  |
| Eosinophils 10^9/L | D0 | 0.21 | ± | 0.05 | 0.15 | - | 0.24 | 0.32 | ± | 0.14 | 0.17 | - | 0.44 | 0.72 |
|  | D56 | 0.23 | ± | 0.25 | 0.06 | - | 0.52 | 0.17 | ± | 0.12 | 0.03 | - | 0.25 |  |
| Basophils 10^9/L | D0 | 0.00 | ± | 0.00 | 0.00 | - | 0.00 | 0.00 | ± | 0.00 | 0.00 | - | 0.00 | 0.65 |
|  | D56 | 0.01 | ± | 0.01 | 0.00 | - | 0.02 | 0.01 | ± | 0.01 | 0.01 | - | 0.02 |  |
| Red blood cell count 10^12/L | D0 | 9.02 | ± | 0.25 | 8.75 | - | 9.24 | 10.38 | ± | 1.89 | 9.14 | - | 12.56 | 0.52 |
|  | D56 | 6.76 | ± | 1.92 | 4.88 | - | 8.71 | 7.97 | ± | 2.24 | 5.40 | - | 9.54 |  |
| Hemoglobin g/L | D0 | 146.00 | ± | 5.00 | 141.00 | - | 151.00 | 147.00 | ± | 5.00 | 142.00 | - | 152.00 | 0.59 |
|  | D56 | 104.67 | ± | 24.01 | 84.00 | - | 131.00 | 118.67 | ± | 32.93 | 81.00 | - | 142.00 |  |
| Mean corpuscular volume (MCV) fL | D0 | 69.23 | ± | 2.89 | 66.10 | - | 71.80 | 66.73 | ± | 2.72 | 64.60 | - | 69.80 | 0.51 |
|  | D56 | 59.37 | ± | 2.35 | 57.50 | - | 62.00 | 61.17 | ± | 3.56 | 57.30 | - | 64.30 |  |
| Mean corpuscular hemoglobin (MCH) pg | D0 | 16.17 | ± | 0.15 | 16.00 | - | 16.30 | 14.43 | ± | 2.39 | 11.70 | - | 16.10 | 0.40 |
|  | D56 | 15.67 | ± | 1.25 | 14.80 | - | 17.10 | 14.90 | ± | 0.00 | 14.90 | - | 14.90 |  |
| Mean corpuscular hemoglobin concentration (MCHC) g/L | D0 | 234.00 | ± | 11.79 | 224.00 | - | 247.00 | 215.67 | ± | 30.17 | 181.00 | - | 236.00 | 0.14 |
|  | D56 | 263.67 | ± | 10.69 | 257.00 | - | 276.00 | 244.67 | ± | 14.19 | 232.00 | - | 260.00 |  |

| **Parameter** | **Day** | **Excipient** | | | | | | **rNGFSP** | | | | | | p-value |
| --- | --- | --- | --- | --- | --- | --- | --- | --- | --- | --- | --- | --- | --- | --- |
|  |  | Mean ± DESVEST | | | MIN - MAX | | | Mean ± DESVEST | | | MIN - MAX | | |  |
| Total protein | D0 | 54.75 | ± | 4.03 | 51.90 | - | 57.60 | 60.37 | ± | 1.96 | 58.30 | - | 62.20 | 0.34 |
|  | D56 | 59.30 | ± | 3.69 | 55.10 | - | 62.00 | 55.87 | ± | 4.07 | 52.30 | - | 60.30 |  |
| Albumin | D0 | 34.30 | ± | 1.27 | 33.40 | - | 35.20 | 36.73 | ± | 0.25 | 36.50 | - | 37.00 | 0.94 |
|  | D56 | 34.47 | ± | 2.25 | 32.30 | - | 36.80 | 34.30 | ± | 2.52 | 31.60 | - | 36.60 |  |
| Globulin | D0 | 20.45 | ± | 2.76 | 18.50 | - | 22.40 | 23.63 | ± | 1.72 | 21.80 | - | 25.20 | 0.34 |
|  | D56 | 24.83 | ± | 3.86 | 20.80 | - | 28.50 | 21.57 | ± | 3.44 | 17.60 | - | 23.70 |  |
| A/G | D0 | 1.70 | ± | 0.14 | 1.60 | - | 1.80 | 1.57 | ± | 0.12 | 1.50 | - | 1.70 | 0.39 |
|  | D56 | 1.40 | ± | 0.26 | 1.10 | - | 1.60 | 1.63 | ± | 0.32 | 1.40 | - | 2.00 |  |
| Total bilirubin | D0 | 3.47 | ± | 1.14 | 2.66 | - | 4.27 | 3.05 | ± | 0.34 | 2.74 | - | 3.42 | 0.68 |
|  | D56 | 3.03 | ± | 1.00 | 2.11 | - | 4.10 | 3.39 | ± | 1.00 | 2.45 | - | 4.44 |  |
| Alanine aminotransferase (ALT) | D0 | 38.50 | ± | 2.12 | 37.00 | - | 40.00 | 38.67 | ± | 8.39 | 29.00 | - | 44.00 | 0.96 |
|  | D56 | 32.67 | ± | 9.07 | 23.00 | - | 41.00 | 33.00 | ± | 2.65 | 31.00 | - | 36.00 |  |
| Aspartate aminotransferase (AST) | D0 | 79.50 | ± | 13.44 | 70.00 | - | 89.00 | 63.33 | ± | 1.53 | 62.00 | - | 65.00 | 0.51 |
|  | D56 | 89.00 | ± | 25.06 | 63.00 | - | 113.00 | 169.33 | ± | 175.56 | 64.00 | - | 372.00 |  |
| AST/ALT ratio | D0 | 2.18 | ± | 0.33 | 1.95 | - | 2.41 | 1.69 | ± | 0.39 | 1.43 | - | 2.14 | 0.54 |
|  | D56 | 2.86 | ± | 1.06 | 1.85 | - | 3.96 | 5.24 | ± | 5.53 | 1.78 | - | 11.62 |  |
| Gamma-glutamyl transferase (GGT) | D0 | 1.15 | ± | 0.78 | 0.60 | - | 1.70 | 1.47 | ± | 0.15 | 1.30 | - | 1.60 | 0.39 |
|  | D56 | 1.03 | ± | 0.85 | 0.20 | - | 1.90 | 4.20 | ± | 5.03 | 1.00 | - | 10.00 |  |
| BUN (Blood Urea Nitrogen) | D0 | 11.46 | ± | 2.61 | 9.61 | - | 13.30 | 10.83 | ± | 0.68 | 10.30 | - | 11.60 | 0.69 |
|  | D56 | 9.35 | ± | 1.92 | 7.47 | - | 11.30 | 8.69 | ± | 1.85 | 6.73 | - | 10.40 |  |
| Creatinine | D0 | 42.50 | ± | 9.19 | 36.00 | - | 49.00 | 40.00 | ± | 5.20 | 37.00 | - | 46.00 | 0.36 |
|  | D56 | 41.67 | ± | 4.51 | 37.00 | - | 46.00 | 38.33 | ± | 3.06 | 35.00 | - | 41.00 |  |
| BUN/Creatinine ratio | D0 | 66.50 | ± | 0.71 | 66.00 | - | 67.00 | 67.33 | ± | 4.73 | 62.00 | - | 71.00 | 0.98 |
|  | D56 | 57.00 | ± | 18.08 | 40.00 | - | 76.00 | 57.33 | ± | 16.50 | 41.00 | - | 74.00 |  |
| Glucose (GLU) | D0 | 8.14 | ± | 2.44 | 6.41 | - | 9.86 | 9.73 | ± | 0.72 | 8.91 | - | 10.26 | 0.35 |
|  | D56 | 6.91 | ± | 0.46 | 6.38 | - | 7.23 | 12.26 | ± | 7.77 | 6.76 | - | 21.15 |  |

**Supplementary Table 3**
